# Supplementary material for: Burden and risk factors of suspected cholangiocarcinoma in high Opisthorchis viverrini endemic rural communities in southern Lao PDR
Source: PLoS Negl Trop Dis. 2024 Nov 27;18(11):e0012617. doi: 10.1371/journal.pntd.0012617 (PMC11602099; doi:10.1371/journal.pntd.0012617)
Supplement: S1 Appendix — (DOCX) [file pntd.0012617.s001.docx]

**Appendix 1: distributions of participants**

| **No** | **Village name** | **Targeted participants** | | | **Complete US examination** | | | **Participants used in analysis** | | |
| --- | --- | --- | --- | --- | --- | --- | --- | --- | --- | --- |
|  |  | **N** | **Female, (%)** | **Mean age (±SD)** | **N** | **Female, (%)** | **Mean age (±SD)** | **N** | **Female, (%)** | **Mean age (±SD)** |
| **Champasack province** | | | | | | | | | | |
| **1** | Donesome | 181 | 93 (51.4) | 49.0 (±11.4) | 60 | 31 (51.7) | 48.4 (±10.9) | 56 | 30 (53.6) | 48.3 (±11.0) |
| **2** | Donekaden | 104 | 59 (56.7) | 51.4 (±11.5) | 93 | 51 (54.8) | 50.7 (±11.0) | 92 | 51 (55.4) | 50.6 (±11.0) |
| **3** | Donekon | 553 | 329 (59.5) | 50.3 (±11.9) | 329 | 198 (60.2) | 50.5 (±11.9) | 318 | 191 (60.1) | 50.5 (±11.8) |
| **4** | Donepheuy | 77 | 44 (57.1) | 50.2 (±12.1) | 47 | 29 (61.7) | 50.9 (±11.4) | 2 | 2 (100.0) | 43.0 (±9.9) |
| **5** | Kaengkoum | 216 | 120 (55.6) | 48.6 (±10.9) | 137 | 88 (64.2) | 48.8 (±10.8) | 129 | 83 (64.3) | 48.6 (±10.8) |
| **6** | Meungsaen | 619 | 320 (51.7) | 49.9 (±11.5) | 322 | 185 (57.5) | 48.1 (±9.7) | 308 | 174 (56.5) | 48.2 (±9.7) |
| **7** | Nangkaud | 198 | 118 (59.6) | 52.8 (±13.3) | 105 | 62 (59.1) | 51.3 (±11.2) | 100 | 58 (58.0) | 51.3 (±11.3) |
| **8** | Phonpeuy | 241 | 141 (58.5) | 50.4 (±12.4) | 133 | 85 (63.9) | 49.1 (±10.6) | 130 | 85 (65.4) | 49.7 (±9.6) |
| **9** | Saenhard-noi | 193 | 101 (52.3) | 50.1 (±11.9) | 86 | 51 (59.3) | 49.7 (±11.7) | 85 | 50 (58.8) | 49.6 (±11.7) |
| **10** | Thaphao | 156 | 91 (58.3) | 47.0 (±10.5) | 98 | 62 (63.3) | 46.3 (±10.2) | 97 | 62 (63.9) | 46.4 (±10.2) |
| **11** | Thapho-tai | 228 | 123 (54.0) | 51.8 (±12.1) | 98 | 50 (51.0) | 50.3 (±10.1) | 96 | 49 (51.0) | 49.9 (±9.7) |
| **12** | Vernsom | 582 | 315 (54.1) | 49.2 (±11.7) | 338 | 216 (63.9) | 49.8 (±10.9) | 298 | 192 (64.4) | 49.8 (±10.9) |
| **Savannakhet province** | | | | | | | | | | |
| **1** | Dongmeung | 258 | 145 (56.2) | 51.9 (±12.3) | 137 | 82 (59.9) | 50.3 (±10.8) | 134 | 80 (59.7) | 50.4 (±10.9) |
| **2** | Nonkoun | 193 | 108 (56.0) | 51.6 (±12.2) | 99 | 49 (49.05) | 99 (±52.1) | 92 | 49 (50.0) | 92 (±52.1) |
| **3** | Paiykhong | 305 | 170 (55.7) | 49.2 (±11.2) | 153 | 89 (58.2) | 49.9 (±10.7) | 151 | 87 (57.6) | 49.7 (±10.6) |
| **4** | Phondok | 234 | 133 (56.8) | 48.3 (±10.5) | 136 | 85 (62.5) | 47.3 (±8.3) | 132 | 83 (62.9) | 47.3 (±8.3) |
| **5** | Phonmaung | 665 | 374 (56.2) | 48.9 (±10.0) | 356 | 224 (62.9) | 49.7 (±9.6) | 344 | 219 (63.7) | 49.1 (±9.6) |
| **6** | Sakeun-neu | 527 | 292 (55.4) | 51.0 (±11.5) | 325 | 198 (60.9) | 50.9 (±11.0) | 323 | 196 (60.7) | 51.0 (±11.0) |
| **7** | Sakeun-tai | 401 | 217 (54.1) | 51.5 (±12.1) | 258 | 157 (60.8) | 52.2 (±11.0) | 252 | 153 (60.7) | 52.3 (±11.1) |
| **8** | Tharmaung | 243 | 130 (53.5) | 52.6 (±23.2) | 177 | 96 (54.2) | 51.9 (±12.5) | 168 | 91 (54.2) | 52.1 (±12.4) |
| **9** | Tharmeung | 276 | 149 (54.0) | 49.9 (±10.9) | 96 | 52 (54.2) | 51.7 (±8.7) | 93 | 50 (53.8) | 51.7 (±8.8) |
| **Total** | | 6,450 | 3,572 (55,4) | 50.2 (±12.2) | 3,583 | 2,140 (59.7) | 50.0 (±10.8) | 3,400 | 2,032 (59.8) | 50.0 (±10.7) |

***Note.*** N represents the number of participations; ±SD indicated the values as mean ± standard deviation
