# Supplementary material for: Burden and risk factors of suspected cholangiocarcinoma in high Opisthorchis viverrini endemic rural communities in southern Lao PDR
Source: PLoS Negl Trop Dis. 2024 Nov 27;18(11):e0012617. doi: 10.1371/journal.pntd.0012617 (PMC11602099; doi:10.1371/journal.pntd.0012617)
Supplement: S2 Appendix — (DOCX) [file pntd.0012617.s002.docx]

**Appendix 2: Multivariate analysis of covariates associated with suspected cholangiocarcinoma among 3,400 study participants (full covariates model)**

| **Covariates** | **Bivariate model** | **Model adjusted for gender and age** | **Model adjusted for all socioeconomic & demographic** | **Model adjusted for co-morbidity** | **Fully adjusted model** |
| --- | --- | --- | --- | --- | --- |
|  | **cOR (95% CI)** | **aOR (95% CI)** | **aOR (95% CI)** | **aOR (95% CI)** | **aOR (95% CI)** |
|  | **Risk factors** | | | | |
| ***Opisthorchis viverrini*** |  |  |  |  |  |
| Negative | Ref | Ref | Ref | Ref | Ref |
| Positive | 3.53 (1.84−6.77)*** | 3.60 (1.87−6.92)*** | 3.39 (1.76−6.53)*** | 3.47 (1.81−6.66)*** | 3.35 (1.74−6.45)*** |
| **Hepatitis B status** |  |  |  |  |  |
| HBsAg- | Ref | Ref | Ref | Ref | Ref |
| HBsAg+ | 1.07 (0.54−2.12) | 1.11 (0.56−2.21) | 1.10 (0.55−2.20) | 1.08 (0.55−2.15) | 1.10 (0.55−2.19) |
| **T2DM** |  |  |  |  |  |
| Non-DM | Ref | Ref | Ref | Ref | Ref |
| DM | 0.69 (0.42−1.14) | 0.63 (0.38−1.05) | 0.65 (0.34−1.08) | 0.80 (0.48−1.32) | 0.73 (0.43−1.22) |
| **Post cholecystectomy** |  |  |  |  |  |
| No | Ref | Ref | Ref | Ref | Ref |
| Yes | 2.46 (1.37−4.41)*** | 2.60 (1.43−4.74)*** | 2.70 (1.48−4.93)*** | 2.66 (1.47−4.83)*** | 2.67 (1.46−4.89)*** |
| **Smoking** |  |  |  |  |  |
| Never smokers | Ref | Ref | Ref | Ref | Ref |
| Past & current | 1.18 (0.90−1.55) | 0.99 (0.73−1.35) | 0.94 (0.69−1.30) | 1.14 (0.86−1.51) | 0.93 (0.68−1.28) |
| **Alcohol consumption** |  |  |  |  |  |
| Less | Ref | Ref | Ref | Ref | Ref |
| ≥ 3 times per week | 1.13 (0.66−1.95) | 1.01 (0.57−1.77) | 1.02 (0.58−1.80) | 1.06 (0.61−1.84) | 1.03 (0.58−1.82) |
|  | **Socioeconomic & demographic** | | | | |
| **Gender** |  |  |  |  |  |
| Male | Ref | Ref | Ref | - | Ref |
| Female | 0.71 (0.55−0.91)** | 0.71 (0.53−0.95)* | 0.65 (0.48−0.89)** | - | 0.67 (0.49−0.91)* |
| **Age groups** |  |  |  |  |  |
| 35-49 y | Ref | Ref | Ref | - | Ref |
| 50-59 y | 1.35 (1.00−1.82)* | 1.39 (1.02−1.89)* | 1.39 (1.02−1.89)* | - | 1.39 (1.02−1.89)* |
| ≥ 60 y | 1.70 (1.23−2.31)*** | 1.66 (1.21−2.28)* | 1.55 (1.10−2.19)* | - | 1.54 (1.09−2.18)* |
| **Province** |  |  |  |  |  |
| Champasack | Ref | - | Ref | - | Ref |
| Savannakhet | 1.14 (0.62−2.11) | - | 0.81 (0.39−1.64) | - | 0.81 (0.40−1.65) |
| **Education** |  |  |  |  |  |
| Illiterate | Ref | - | Ref | - | Ref |
| Up to primary school | 0.89 (0.63−1.25) | - | 0.93 (0.64−1.33) | - | 0.93 (0.64−1.34) |
| Secondary school & above | 0.69 (0.48−1.00) | - | 0.74 (0.49−1.13) | - | 0.75 (0.49−1.14) |
| **Profession** |  |  |  |  |  |
| House wife/elderly/retired | Ref | - | Ref | - | Ref |
| Farmer/labour/fishermen | 0.94 (0.61−1.45) | - | 1.12 (0.71−1.77) | - | 1.14 (0.72−1.81) |
| Civil servant/trader | 0.52 (0.28−0.97)* | - | 0.71 (0.36−1.37) | - | 0.72 (0.37−1.39) |
| **Socio-economic status** |  |  |  |  |  |
| Poor tertile | Ref | - | Ref | - | Ref |
| Less tertile | 1.11 (0.70−1.75) | - | 1.18 (0.72−1.95) | - | 1.19 (0.72−1.97) |
| Least tertile | 1.36 (0.83−2.23) | - | 1.45 (0.82−2.57) | - | 1.46 (0.82−2.58) |
|  | **Co-morbidity** | | | | |
| **Fatty liver** |  |  |  |  |  |
| Absence or mild | Ref | - | - | Ref | Ref |
| Moderate or severe | 0.42 (0.24−0.74)** | - | - | 0.47 (0.27−0.83)** | 0.49 (0.28−0.86)* |
| **Obesity** |  |  |  |  |  |
| Lean | Ref | - | - | Ref | Ref |
| Not lean | 0.84 (0.65−1.08) | - | - | 0.94 (0.72−1.23) | 0.98 (0.75−1.27) |

***Notes*.** CI: confidence intervals; cOR crude odds ratio; aOR: adjusted odds ratio; T2DM: type 2 diabetes mellitus; HBsAg: hepatitis B virus surface antigen; PZQ: Praziquantel. *p-value < 0.05, **p-value < 0.01, ***p-value < 0.001; Ref: reference.
