# Supplementary material for: Burden and risk factors of suspected cholangiocarcinoma in high Opisthorchis viverrini endemic rural communities in southern Lao PDR
Source: PLoS Negl Trop Dis. 2024 Nov 27;18(11):e0012617. doi: 10.1371/journal.pntd.0012617 (PMC11602099; doi:10.1371/journal.pntd.0012617)
Supplement: S3 Appendix — (DOCX) [file pntd.0012617.s003.docx]

**Appendix 3: Sensitivity analysis**

| **Covariates** | **Exclude individuals with T2DM** | **Exclude individuals with heavy OV infection intensity** | **Exclude individuals who has cholecystectomy done** |
| --- | --- | --- | --- |
|  | **aOR (95% CI)** | **aOR (95% CI)** | **aOR (95% CI)** |
|  | **Risk factors** | | |
| ***Opisthorchis viverrini*** |  |  |  |
| Negative | Ref | Ref | Ref |
| Positive | 3.41 (1.71−6.80)*** | 3.00 (1.54−5.78)*** | 3.91 (1.89−8.10)*** |
| **Hepatitis B status** |  |  |  |
| HBsAg- | Ref | Ref | Ref |
| HBsAg+ | 1.15 (0.57−2.31) | 0.99 (0.46−2.13) | 1.13 (0.56−2.26) |
| **T2DM** |  |  |  |
| Non-DM | - | Ref | Ref |
| DM | - | 0.78 (0.45−1.35) | 0.71 (0.41−1.22) |
| **Post cholecystectomy** |  |  |  |
| No | Ref | Ref | - |
| Yes | 2.54 (1.33−4.86)*** | 2.76 (1.44−5.27)*** | - |
| **Smoking** |  |  |  |
| Never smokers | Ref | Ref | Ref |
| Past & current | 0.91 (0.66−1.27) | 1.01 (0.71−1.43) | 0.95 (0.69−1.30) |
| **Alcohol consumption** |  |  |  |
| Less | Ref | Ref | Ref |
| ≥ 3 times per week | 0.85 (0.46−1.58) | 1.03 (0.54−1.96) | 1.00 (0.57−1.77) |
|  | **Socioeconomic & demographic** | | |
| **Gender** |  |  |  |
| Male | Ref | Ref | Ref |
| Female | 0.67 (0.49−0.92)** | 0.64 (0.45−0.90)*** | 0.67 (0.49−0.91)* |
| **Age groups** |  |  |  |
| 35-49 y | Ref | Ref | Ref |
| 50-59 y | 1.45 (1.05−1.99)* | 1.42 (1.01−2.00)* | 1.38 (1.00−1.90)* |
| ≥ 60 y | 1.46 (1.02−2.10)* | 1.52 (1.03−2.23)* | 1.54 (1.08−2.20)* |
| **Province** |  |  |  |
| Champasack | Ref | Ref | Ref |
| Savannakhet | 0.87 (0.42−1.79) | 1.08 (0.52−2.25) | 0.82 (0.40−1.67) |
| **Education** |  |  |  |
| Illiterate | Ref | Ref | Ref |
| Up to primary school | 1.00 (0.68−1.47) | 0.94 (0.63−1.39) | 0.94 (0.65−1.38) |
| Secondary school & above | 0.78 (0.50−1.20) | 0.71 (0.45−1.12) | 0.79 (0.51−1.21) |
| **Profession** |  |  |  |
| House w./elderly/retired | Ref | Ref | Ref |
| Farmer/laborer/fishermen | 1.18 (0.72−1.94) | 0.98 (0.60−1.61) | 1.12 (0.69−1.82) |
| Civil servant/trader | 0.77 (0.38−1.57) | 0.66 (0.32−1.35) | 0.75 (0.38−1.50) |
| **Socio-economic status** |  |  |  |
| Poor tertile | Ref | Ref | Ref |
| Middle tertile | 1.16 (0.69−1.95) | 1.03 (0.57−1.84) | 1.15 (0.69−1.91) |
| Wealthy tertile | 1.41 (0.78−2.54) | 1.24 (0.64−2.37) | 1.45 (0.81−2.60) |
|  | **Co-morbidity** | | |
| **Fatty liver** |  |  |  |
| Absence & mild | Ref | Ref | Ref |
| Moderate & severe | 0.48 (0.26−0.91)* | 0.53 (0.29−0.96)* | 0.49 (0.27−0.89)* |
| **Obesity** |  |  |  |
| Lean | Ref | Ref | Ref |
| Not lean | 0.96 (0.73−1.27) | 1.05 (0.79−1.41) | 0.94 (0.71−1.24) |

***Notes*.** CI: confidence intervals; aOR: adjusted odds ratio; OV *Opisthorchis viverrini*; DM diabetes mellitus; HBsAg hepatitis B virus surface antigen; PZQ - Praziquantel. *p-value < 0.05, **p-value < 0.01, ***p-value < 0.001. House w. – housewife
