# Supplementary material for: Burden and risk factors of suspected cholangiocarcinoma in high Opisthorchis viverrini endemic rural communities in southern Lao PDR
Source: PLoS Negl Trop Dis. 2024 Nov 27;18(11):e0012617. doi: 10.1371/journal.pntd.0012617 (PMC11602099; doi:10.1371/journal.pntd.0012617)
Supplement: S4 Appendix — (DOCX) [file pntd.0012617.s004.docx]

**Appendix 4: individual’s clinical symptoms and liver biochemistry results**

| **Variables** | | **Overall (N=3,400)** |  | **Suspected CCA** | | **No suspected CCA** | | |
| --- | --- | --- | --- | --- | --- | --- | --- | --- |
|  |  | **N (%)** |  | **N=274** | **(%)** | **N=3,126** | | **(%)** |
|  |  | **Clinical symptoms** | | | | | | |
| **Fever** | ≤37.5℃ | 3,388 (99.7) |  | 272 | 8.0 | | 3,116 | 92.0 |
|  | >37.5℃ | 12 (0.4) |  | 2 | 16.7 | | 10 | 83.3 |
| **Jaundice** | Absence | 3,151 (92.7) |  | 255 | 8.1 | | 2,896 | 91.9 |
|  | Presence | 249 (7.3) |  | 19 | 7.6 | | 230 | 92.4 |
| **Spider angioma** | Absence | 3,385 (99.6) |  | 274 | 8.1 | | 3,111 | 91.2 |
|  | Presence | 15 (0.4) |  | 0 | 0.0 | | 15 | 100.0 |
| **Venous collateralis** | Absence | 3,398 (99.9) |  | 274 | 8.1 | | 3,124 | 91.9 |
|  | Presence | 2 (0.1) |  | 0 | 0.0 | | 2 | 100.0 |
| **Right upper quadrant pain (Murphy’s sign)** | Absence | 3,398 (99.7) |  | 273 | 8.1 | | 3,116 | 91.9 |
|  | Presence | 11 (0.3) |  | 1 | 9.1 | | 10 | 90.9 |
| **Abdominal ascites** | Absence | 3,392 (99.8) |  | 274 | 81 | | 3,118 | 91.9 |
|  | Presence | 8 (0.2) |  | 0 | 0.0 | | 8 | 100.0 |
| **Palpable liver edge at deep inspiration** | Presence | 3,314 (97.5) |  | 264 | 8.0 | | 3,050 | 92.0 |
|  | Absence | 86 (2.5) |  | 10 | 11.6 | | 76 | 88.4 |
| **Peripheral edema** | Presence | 3,386 (99.6) |  | 273 | 8.1 | | 3,113 | 91.9 |
|  | Absence | 14 (0.4) |  | 1 | 7.1 | | 13 | 92.9 |
|  |  | **Liver biochemistry indicators** | | | | | | |
|  |  | **Mean (SD)** |  | **Mean** | **SD** | | **Mean** | **SD** |
| **AST** |  | 34.6 (27.3) |  | 38.6 | 29.3 | | 34.3 | 27.1 |
| **ALT** |  | 19.9 (16.5) |  | 23.7 | 24.5 | | 19.6 | 15.6 |
| **ALP** |  | 114.7 (62.1) |  | 134.7 | 81.2 | | 112.9 | 59.9 |
| **GGT** |  | 96.1 (133.4) |  | 128.7 | 173.0 | | 93.2 | 129.0 |
| **T Bilirubin** |  | 0.5 (0.6) |  | 0.5 | 0.3 | | 0.5 | 0.6 |
| **Direct Bilirubin** |  | 0.3 (0.2) |  | 0.3 | 0.2 | | 0.3 | 0.2 |

***Notes*. AST-** Aspartate transaminase **; ALT-** Alanine transaminase**; ALP-** Alkaline phosphatase**; GGT-** Gamma-glutamyl transferase; **T** **Bilirubin** – total bilirubin; **D bilirubin**- direct Bilirubin.
